# Frailty as a Predictor of Hospitalization and Low Quality of Life in Geriatric Patients at an Internal Medicine Outpatient Clinic: A Cross-Sectional Study

**DOI:** 10.3390/geriatrics7050089

**Published:** 2022-08-31

**Authors:** Panita Limpawattana, Chudapha Khammak, Manchumad Manjavong, Apichart So-ngern

**Affiliations:** 1 Division of Geriatric Medicine, Department of Internal Medicine, Faculty of Medicine, Khon Kaen University, Khon Kaen 40002, Thailand; 2 Division of Sleep Medicine, Department of Medicine, Faculty of Medicine, Khon Kaen University, Khon Kaen 40002, Thailand

**Keywords:** frail, frail olders, functionally impaired, older adult

## Abstract

Frailty is an aging-associated state that increases patients’ vulnerability to disease, and can lead to various adverse outcomes. It is classified as either physical frailty alone or physical frailty in combination with cognitive impairment (cognitive frailty). There are currently limited data available regarding the prevalence and adverse outcomes of frailty in Thailand. This was a cross-sectional study aimed at determining the prevalence of physical and cognitive frailty and their effects on hospitalization and quality of life. Participants were older patients who attended an internal medicine outpatient clinic. Frailty was diagnosed using the Thai Frailty Index. The Thai version of the MoCA was used to evaluate cognitive status. Univariate and multivariate analyses were performed to compare adverse outcomes in terms of poor quality of life and history of admission to hospital between patients with frailty and non-frail patients, and among patients with physical frailty, cognitive frailty, cognitive impairment, and robust (non-frail and non-cognitively impaired) patients. We enrolled 198 participants. The prevalence of physical and cognitive frailty was 28.78% and 20.70%, respectively. When compared with non-frail patients, frailty was associated with hospitalization (adjusted OR 3.01, *p =* 0.002) but was not significantly related to quality of life (adjusted OR = 1.98, *p* = 0.09). However, physical and cognitive frailty were associated with fair quality of life when compared with normal patients (adjusted OR = 4.34, *p* = 0.04 and adjusted OR = 4.28, *p* = 0.03, respectively). The prevalence of frailty—particularly cognitive frailty—was high. Frailty was associated with adverse outcomes in terms of hospitalization and quality of life.

## 1. Introduction

Frailty is a geriatric syndrome that that can lead to vulnerability and suffering in older adults. Patients with frailty are more likely to have a lower physiologic reserve than normal older adults [1], possibly leading to poor outcomes after conditions such as infection and trauma.

Previous studies have found frailty to be associated with various diseases such as cardiovascular disease, diabetes mellitus, COPD, asthma, stroke, foot pain, cognitive impairment, and depression [2,3,4,5]. These comorbidities are usually found in older people, so frailty status should be evaluated in these patients. Moreover, frailty could increase the risk of several adverse outcomes such as hospitalization, prolonged hospital stay, readmission after discharge, falling, dependency, decreased quality of life, and mortality [6,7,8,9,10].

Despite lacking a gold standard for diagnosing frailty, several criteria or assessment methods have been developed to define and identify the condition such as the phenotypic definition of frailty, the frailty index, the FRAIL scale, and the Edmonton frailty scale [11,12,13,14]. In previous studies, the prevalence of frailty in older adults has varied from 4 to 59.1%, depending on the frailty assessment method used and the geographic region [15,16,17]. In addition, some factors such as nutritional status (both low body weight and obesity), low medication adherence, and gender might influence frailty status [17,18]. A systematic review of a community-based cohort found that the prevalence of frailty was higher in women than in men [17]. However, the results of a prior study in a Spanish population showed that gender did not affect frailty status in older patients with foot pain [19].

The prevalence of frailty was explored in many countries. For example, a study from Australia found the prevalence of frailty was 15.2% based on the FRAIL scale [20]. A study from Taiwan determined frailty by using the Fried Frailty Index and found that the prevalence of frailty in community-dwelling Taiwanese was 4.9% [21]. Another previous study in Thailand (an aging society) found the prevalence of frailty in a community setting to be 22.1%, based on the Thai Frailty Index (TFI) [15]. Therefore, different cultures and socioeconomic status might have an effect on the prevalence of frailty.

Additionally, many studies have found frailty to be closely related to cognitive impairment. The term “cognitive frailty” has been proposed as a new geriatric syndrome that describes a combination of physical frailty and mild cognitive impairment (MCI; a condition between normal aging and dementia characterized by cognitive impairment that is not severe enough to impact activities of daily living) [22,23]. Older patients with cognitive frailty have a higher risk of mortality and adverse outcomes compared with their healthy counterparts or patients with physical frailty alone. The reason is that MCI alone could impact patients’ quality of life because a patient with impaired cognition might feel insecure about living independently [24], so MCI in combination with frailty (cognitive frailty) might have higher risk of adverse outcomes than physical frailty alone.

The prevalence of cognitive frailty is still unclear. The results of a systematic review and meta-analysis showed that the estimated prevalence of cognitive frailty might be 6% or 16% [25]. Although previous studies have found the prevalence of cognitive frailty in the Thai community to be about 3–5% [26,27,28], the prevalence might be lower than estimated. This hypothesis could be explained by how participants in prior studies were evaluated regarding their cognitive status by using the Mini Mental Status Examination (MMSE), but this tool was not appropriate for distinguishing MCI patients from normal older adults [29,30]. Data on the differences and a comparison of the adverse outcomes between robust people and those with cognitive and physical frailty are limited.

As mentioned above, frailty is associated with many diseases, so older patients in an ambulatory medicine department who usually have many comorbidities may have a high prevalence of frailty. However, its prevalence and adverse outcomes in ambulatory settings in Thailand have not been examined.

Therefore, the primary objective of this study was to determine the prevalence of frailty in older patients in an internal medicine outpatient clinic. The secondary objective was to determine the prevalence of cognitive frailty and to compare related hospitalization and quality-of-life outcomes in the studied population.

## 2. Materials and Methods

### 2.1. Participants and Setting

This study was part of a research project entitled “Prevalence of Frailty and Associated Adverse Events in Older Patients of an Outpatient Medicine Clinic: A Cross-sectional Study”. Data were collected between 1 October 2019 and 31 January 2022 at the internal medicine outpatient clinic of Khon Kaen University Srinagarind Hospital (Thailand). Participants who met the following criteria were enrolled: (1) age ≥ 60 years and (2) having attended Srinagarind Hospital’s Internal Medicine outpatient clinic. The study excluded older patients who fit any of the following criteria: (1) inability to speak the Thai language, (2) dementia or acute psychosis, (3) aphasia with an inability to communicate with others, (4) inability to stand upright or acute arthritis, (5) acute infection, or (6) severe visual impairment.

### 2.2. Operational Definitions

**Frailty**: The Thai Frailty Index (TFI), which has been validated in the Thai population, was used to identify frailty in older adults. This tool consists of 30 variables to determine current health status, medical comorbidities, and emotional and physical function. The total possible score is 1, calculated as the number of items that apply to the patient (defined as deficits) divided by 30 [15]. Older adults with a TFI greater than 0.25 are considered to have frailty.

**Cognitive frailty** was defined as the coexistence of physical frailty and mild cognitive impairment (MCI) [31]. Patients with MCI usually present with the following signs: (1) concern regarding a change in cognition on the part of the patient, someone who knows the patient well, or a skilled clinician observing the patient; (2) evidence of lower performance than what would be expected for the patient’s age and educational background in one or more cognitive domains; (3) preservation of independence in functional abilities; and (4) no evidence of dementia.

For this study, the Thai version of Montreal Cognitive Assessment test (MoCA-T) was used to evaluate the participants’ cognitive status. A previous study conducted at the Internal Medicine outpatient clinic of Srinagarind Hospital revealed that a MoCA-T score < 20 was an appropriate cut-off point to indicate MCI in older patients in this setting, with a sensitivity of 0.76 and specificity of 0.71 [32].

**Physical frailty** was defined as meeting the criteria of frailty (TFI > 0.25) with a MoCA-T score ≥ 20.

**Robust patient** refers to participants who were non-frail (TFI ≤ 0.25) and non-cognitively impaired (MoCA-T ≥ 20).

**Quality of life** was determined based on the Thai version of the World Health Organization Quality of Life Brief—Old (WHOQOL-Old), a self-report questionnaire with a satisfactory level of internal consistency (Cronbach’s alpha coefficient = 0.8) [33]. The questionnaire contains 24 items divided into six domains: (1) perceptive ability, (2) autonomy, (3) social activity, (4) intimacy, (5) activities of daily living, and (6) fear of death. The score ranges from 24–120. A score of 24–55 indicates poor quality of life, 56–88 indicates fair quality of life, and 89–120 indicates good quality of life.

**Depression**: Participants’ depressive status was evaluated using the Thai version of the Patient Health Questionnaire-9 [PHQ-9]. This depression screening tool consists of nine items with a total score of 27. A cut-off score of ≥9 has a sensitivity of 0.84 and specificity of 0.77 [34].

**Adverse events**: Adverse events in this study included poor and fair quality of life and a history of admission within the past year.

### 2.3. Data Collection

This study was approved by the Khon Kaen University Faculty of Medicine Ethics Committee for Human Research in accordance with the Declaration of Helsinki. All participants provided written informed consent prior to data collection. This study is reported following the STROBE guidelines [35].

Demographic data, including age, sex, education level, marital status, number of family members, household income, underlying diseases, number of medications used, sedative drug use, depression, number of hospitalizations within one year, and tobacco consumption, were collected from patient medical records and the questionnaires. Subsequently, a trained clinical researcher administered the TFI, WHOQOL-Old, PHQ-9, and MoCA-T.

### 2.4. Statistical Analysis

Regarding the demographic data, categorical variables were presented as numbers and percentages, whereas continuous variables were presented as means and standard deviations. If the data were not normally distributed, the median and interquartile range were used instead. The prevalence of frailty and cognitive frailty were presented as percentages. Quality of life and history of admission within one year were compared between frail and non-frail patients, and between frail and normal patients. Univariate analysis was used to assess the proportional differences in the categorical variables between the two groups of participants. The results were presented as crude odds ratios (ORs) with a 95% confidence interval (CI). A *p*-value < 0.05 was considered to indicate a statistically significant difference. For multivariate analysis, the results were adjusted for depression, marital status, income, age, and education level. The adjusted odds ratio (AOR) and 95% CI were used to determine the effect size. Missing data were analyzed as missing. Data analysis was performed using R version 4.1.2.

### 2.5. Sample Size Calculation

The formula for estimating an infinite population proportion was used to estimate the sample size. Based on a previous study in Japan, the prevalence of frailty in an ambulatory setting was estimated to be 24% [36]. With an estimated error of about 6%, the required sample size for this study was 198.

## 3. Results

### 3.1. Participant Characteristics and Prevalence of Frailty

One hundred ninety-eight patients were enrolled in this study, 124 (62.63%) of whom were female. The prevalence of frailty was 28.78% (57/198). Of the 57 frail participants, 41 were determined to suffer from cognitive frailty, making the overall prevalence of physical and cognitive frailty 8.08% (16/198) and 20.7% (41/198), respectively.

Demographic and clinical data are presented in Table 1. Frail participants were more likely to be older and have comorbidities than those in the non-frail group. With regard to socioeconomic status, most of the patients with frailty had fewer than 6 years of education (77.19%) and had a household income less than THB20,000/month (89.47%).

### 3.2. Adverse Events Related to Physical and Cognitive Frailty

#### 3.2.1. Comparison between Frail and Non-Frail Participants

Table 2 compares related adverse events between frail and non-frail older adults. No participants in this study had poor quality of life, but a higher percentage of patients with frailty had fair quality of life compared with those in the non-frail group (OR 2.70 (1.32–5.54), *p* = 0.01). However, these results were not statistically significant after adjustment for depressive status, marital status, income, age, education level, and history of admission. The frail group had a significantly higher rate of hospitalization within the past year, with an AOR of 3.01 (1.51–6.12, *p* = 0.002).

#### 3.2.2. Comparison of History of Admission among Participants, Depending on Frail and Cognitive Status

A comparison of related adverse events among robust, physical frail, and cognitive frail participants is presented in Table 3. The patients with frailty (both physical and cognitive frailty) had poorer quality of life than those in the robust group, with AOR 4.34 [1.02–17.7], *p* = 0.04 and AOR 4.28 [1.18–16.59], *p* = 0.03, respectively. Conversely, a history of admission within the past year did not differ significantly between groups.

## 4. Discussion

Frailty is a disorder frequently found in older adults. It not only reduces their ability to cope with stress but also increases the risk of unwanted complications such as mental and physical illness. The prevalence of frailty varies by geographic region, socioeconomic background, and the assessment tools used. This study found that older patients who came to the outpatient department at Srinagarind Hospital had a frailty prevalence of 28.78%, with physical frailty accounting for 8.08% and cognitive frailty accounting for 20.70%. The prevalence of frailty in this study was higher than the 22.1% found in a previous study conducted at Siriraj Hospital of Mahidol University in Bangkok, Thailand, which used the Thai frailty index to study patients aged >60 years in a community setting [15]. In the present study, most of the patients had multiple risk factors for frailty such as chronic disease, polypharmacy, and frequent hospital stays. Furthermore, other factors, such as socioeconomic status, education, culture, and degree of social support, can also affect frailty status.

Cognitive frailty is defined as the coexistence of frailty and mild cognitive impairment. This study found cognitive frailty in 20.70% of participants. This is much higher than the 3–5% prevalence found in another study conducted at Siriraj Hospital in Bangkok [28]. There are several possible reasons for this difference. One is that the prior study used the modified MINI-COG-PS to detect cognitive impairment, while we used the Thai version of the MoCA, which may have a higher sensitivity for detecting cognitive impairment in older patients. Prior studies conducted in Srinagarind Hospital found that a MoCA score < 20 can diagnose MCI in an outpatient population with 76.2% sensitivity and 71.3% specificity, while the Mini-COG provided only 69% sensitivity and 73% specificity [32,37]. Another possible reason for this discrepancy may be the differences in the studied populations. For example, most of the patients in our study had fewer than 6 years’ education, a household income less than THB20,000/month, and multiple underlying diseases. These factors indicate that socioeconomic differences may play a role in the prevalence of cognitive frailty. Moreover, our study showed that the prevalence of cognitive frailty in patients with frailty was 71.92%. This result could encourage physicians to conduct cognitive evaluations in patients with frailty.

We found that a greater percentage of patients with frailty had a fair quality of life compared with non-frail patients (33.33% vs. 15.60%). This is consistent with a previous study conducted in a group of patients with frailty in a geriatric medicine outpatient clinic in Italy, which used the OPQOL questionnaire to assess quality of life and found that frailty was associated with poor quality of life [38]. This may be because frailty tends to negatively impact all four quality-of-life domains (physical, psychological, social relationships, and environment). However, the association between frailty and quality of life in the current study was not statistically significant after adjustment for other variables (*p* = 0.09). This may be because some of the non-frail participants in this study had cognitive impairment, which could result in poorer quality of life [39]. However, the difference in quality of life was significant when compared between robust patients (non-frail and non-MCI) and patients with frailty (physical frailty: *p* = 0.04; cognitive frailty: *p* = 0.03).

Compared with robust participants, our results showed that patients with cognitive frailty had a lower risk of fair quality of life than patients with physical frailty alone (AOR 4.28 and 4.34, consequently). These results slightly differed from our expectation because patients with both conditions (impaired cognition and physical frailty) should have poorer quality of life than patients with physical frailty alone. Our results might be explained by a previous study which explored the association between MCI and quality of life. The author emphasized that MCI decreased patients’ quality of life in terms of autonomy, which meant that MCI patients felt nervous about their cognitive and functional worsening in the future would affect their ability to make decisions, but MCI did not impact to other facets of quality of life such as sensory abilities, intimacy, and social participation [24]. In the current study, the results might be different if we specifically explored the facets of quality of life.

In addition, we found that frailty increased the risk of hospitalization, which is consistent with the findings of several previous studies [40,41,42]. For example, a meta-analysis in chronic heart failure patients showed that frailty was a predictor of all-cause mortality and hospitalization [40]. Another study from Brazil found an association between frailty and hospital or nursing home admission [41]. Older patients with frailty are more likely to be hospitalized due to decreases in their physiologic reserves and deterioration in their overall body function. Consequently, their clinical status is likely to worsen when they are faced with stress [43].

This study had several limitations. One is that it was conducted at an outpatient clinic, so the results might not be generalizable to other settings. Another is that this study did not explore the facet of quality of life deeply. In addition, some factors, such as reasons for hospitalization, were not evaluated. Further studies should thus be conducted to confirm these results.

## 5. Conclusions

This study showed a high prevalence of frailty—especially cognitive frailty—among older patients in an outpatient clinic. Frailty (both physical and cognitive frailty) is associated with adverse events, so frailty screening might help predict outcomes in terms of hospitalization and quality of life.

## Figures and Tables

**Table 1:** Demographic data of the studied population.

Baseline Characteristics	Frail *n* = 57	Non-Frail *n* = 141	*p* Value
Sex, *n* (%)			
Female, *n* (%)	36 (63.15)	88 (62.41)	1
Male, *n* (%)	21 (36.84)	53 (37.59)	
Age (years), med (IQR)	71 (67, 74)	67 (63, 70)	0.06
BMI < 18.5 (kg/m^2^), *n* (%)	4 (7.02)	9 (6.38)	1
Years of education, *n* (%)			
<6	44 (77.19)	69 (48.94)	<0.01
≥6	13 (22.81)	72 (51.06)	
Marital status, *n* (%)			
Married	36 (63.16)	93 (65.96)	0.83
Other *	21 (36.84)	48 (34.04)	
Family members, *n* (%)			
None	4 (7.02)	9 (6.38)	1
>1 member	53 (92.98)	132 (93.62)	
Household income/month, *n* (%)			
≤20,000 baht	51 (89.47)	100 (70.92)	<0.001
>20,000 baht	6 (10.53)	61 (29.08)	
Underlying disease, *n* (%)			
Hypertension	48 (84.21)	80 (56.74)	<0.001
Diabetes mellitus	27 (47.37)	46 (32.62)	0.07
Stroke	10 (17.54)	6 (4.26)	0.003
COPD/asthma	5 (8.77)	7 (4.96)	0.33
CKD	20 (35.09)	14 (9.93)	<0.001
Depression, *n* (%)	8 (14.04)	4 (2.84)	0.06
Falls within 6 months, *n* (%)	12 (21.05)	16 (11.34)	0.12
Polypharmacy, *n* (%)	33 (57.89)	60 (42.55)	0.07
Current alcohol consumption, *n* (%)	5 (8.74)	21 (14.89)	0.35
Current/previous smoking, *n* (%)	17 (29.83)	27.66)	0.89
History of admission within past year, *n* (%)	30 (52.63)	39 (27.65)	0.002
Quality of life, *n* (%)			
Good	38 (66.67)	119 (84.39)	0.01
Fair	19 (33.33)	22 (15.6)	
Poor	0	0	

**Note**: *n*: total number of participants, med: median, IQR: interquartile range, BMI: body mass index (obese, 25 kg/m^2^; overweight, 23–24.9 kg/m^2^; normal 18.5–22.9 kg/m^2^; underweight < 18.5 kg/m^2^), HT: hypertension, DM: diabetes mellitus, CVA: cerebrovascular accident, COPD: chronic obstructive pulmonary disease, CKD: chronic kidney disease. Depression was defined as a Patient Health Questionnaire (PHQ)-9 score ≥ 9. * Other marital statuses included single, divorced, and widowed.

**Table 2:** Comparison of related adverse events between frail and non-frail older adults.

Related Adverse Events	Frail *n*, (%)	Non-Frail *n*, (%)	Crude OR (95%CI)	*p* Value	AOR * (95% CI)	*p* Value
Fair quality of life	19 (33.33)	22 (15.60)	2.70 (1.32–5.54)	0.01	1.98 (0.88–4.43)	0.09
Admission within 1 year	30 (52.63)	39 (27.65)	2.91 (1.54–5.53)	0.001	3.01 (1.51–6.12)	0.002

Note: *n*: total number of participants, AOR: adjusted odds ratio, 95% CI: 95% confidence interval. * Adjusted for depression, marital status, income, age, and education level.

**Table 3:** Comparison of adverse outcomes among participants, depending on frailty and cognitive status.

	History of Admission within Past Year					Fair Quality of Life				
	*n* (%)	COR (95%CI)	*p* Value	AOR * (95%CI)	*p* Value	*n* (%)	COR (95%CI)	*p* Value	AOR * (95%CI)	*p* Value
Robust	24 (29.6)	Ref	-	-	-	7 (8.64)	Ref	-	-	-
Physical frailty	9 (56.3)	3.05 (1.02–9.5)	0.04	2.75 (0.9–8.9)	0.08	5 (31.3)	4.81 (1.2–17.9)	0.019	4.34 (1.02–17.7)	0.04
Cognitive frailty	21 (51.2)	2.49 (1.2–5.5)	0.02	2.28 (2.8–6.6)	0.12	14 (34.2)	5.48 (2.1–15.9)	0.001	4.28 (1.17–16.9)	0.03

Note: *n*: total number of participants, COR: crude odds ratio, AOR: adjusted odds ratio, 95% CI: 95% confidence interval. * Adjusted for depression, marital status, income, age, and education level.

## Data Availability

No new data were created or analyzed in this study. Data sharing is not applicable to this article.
